# Preventive insights and practices of female health professionals regarding cervical cell dysplasia: a cross-sectional study in Egypt

**DOI:** 10.1186/s12889-025-24004-4

**Published:** 2025-11-06

**Authors:** Mira M. Abu-Elenin, Marwa A. Shahin, Doaa E. Abdeldaim

**Affiliations:** 1 Department of Public Health and Community Medicine, Faculty of Medicine, Tanta University, 4th floor, Medical Campus, Al-Giesh St, Tanta, Egypt; 2 Department of Maternal and Newborn Health Nursing, Faculty of Nursing, Menoufia University, Shebin Elkom, Egypt; 3 Nursing Program, Batterjee Medical College, Jeddah, 21442 Saudi Arabia

**Keywords:** Cancer cervix, Female, Health professionals, Screening, Pap smear

## Abstract

**Background:**

Cervical dysplasia is preventable through screening methods and Human Papillomavirus (HPV) vaccination. Cervical cancer (CC) mortality is disproportionately higher in low-and-middle-income nations, which lack a population-based screening program. Health professionals should promptly counsel and educate females about cervical dysplasia prevention.

**Aim:**

this work aimed to determine the level of knowledge, attitudes, and practices of female health professionals about cervical cell dysplasia. As well as addressing the potential barriers against routine cytological screening tests.

**Methods:**

A cross-sectional multicentric study at two tertiary hospitals; Tanta and Menoufia University Hospitals, recruited 1300 women (physicians, nurses, pharmacists, and dentists) via a multistage stratified random sampling technique. A self-administered questionnaire consisting of 4 sections was used to collect the relevant data.

**Results:**

Across all professions, 25% and 49.2% respectively had good knowledge levels and positive attitudes regarding cervical dysplasia. The majority had not been vaccinated against HPV nor undergone a Pap smear. Older age, urban residence, and positive family history were significant predictors of negative attitudes towards screening, *p* < 0.0001, beta 95%(CI) = -0.8 ( -0.1,-0.05), -0.9 (-1.2,-0.6), -0.2 (-0.8,0.4)) respectively. The most encountered barriers opposing screening included lack of awareness about health facilities providing CC screening and the belief that there is no need so far, no complaints (72.6%,73%).

**Conclusion:**

Female health professionals possessed acceptable knowledge and relatively positive attitudes regarding CC prevention, while their practices were discouraging. Believing that CC is a curse was the main culprit of refraining screening. It is pivotal to enhance accessibility to cervical screening services in various healthcare settings and boost the knowledge of health practitioners as they are key promoters of public health.

**Supplementary Information:**

The online version contains supplementary material available at 10.1186/s12889-025-24004-4.

## Introduction

Malignant cervical cell disorder is one of the threatened gynecological tumors, it ranks as the fourth most prevalent type of cancer among women aged between 15 and 44 years worldwide, and the 13th most frequent cancer among women in Egypt [1, 2]. Sexually transmitted viruses like HPV genotypes 16 and 18, Chlamydia, prolonged oral contraceptive use, tobacco smoking, compromised immune system, and low socioeconomic status are potential threats [3]. High parties with multiple pregnancies and multiple sexual partners traumatize the cervix, accelerating HPV infection. Pregnancy hormones may also induce oncogenic activity [4]. Since 70% of cervical cell disorders are HPV-related [5], the HPV vaccine is potentially a preventive measure that could provide long-term immunity against high-risk and low-risk HPV sub-strains and a low level of cross-immunity [6, 7].

Factors of refraining from administering the HPV vaccine include (i) lack of women’s awareness about the importance of the HPV vaccine on cancer prevention. (ii) cultural beliefs (iii) unavailability of HPV vaccine, and (iv) lack of recommendations from the health care practitioners [5, 8, 9].

Cervical dysplasia is asymptomatic in its initial stages, easily treatable, and preventable due to the availability of effective HPV vaccination and screening techniques [5, 10]. Consequently, an early diagnosis is essential for a more advantageous outcome. The Papanicolaou (Pap) smear screening procedure identifies cytological abnormalities in the cervix and greatly enhances the likelihood of early and effective treatment and survival [11].

Many developed countries use molecular testing for high-risk HPV DNA (hr-HPV). This strategy has gained popularity for women over 25 or 30 and is currently recommended as the primary screening tool or co-test with cytology. hrHPV has a higher sensitivity than the liquid-based cytology for early distinguishing of cervical intraepithelial neoplasia (CIN) 2 + or worse (CIN) 3+. Whereas, Pap cytology is highly specific, since it concerns abnormal (pre-malignancies) cells with liable test subjectivity and relatively low sensitivity. Thus, it is used to triage women who test positive in the initial screening [12].

Approximately 85% of cancer cervix-related deaths occur in developing countries, where a lack of screening facilities, limited access to proper healthcare, and poor awareness make early detection of dysplastic changes a serious concern for healthcare professionals [13, 14], resulting in overwhelmed cervical cancer complications prospectively [15].

In Egypt, approximately 36.7 million women aged fifteen years and older are at risk of developing cervical cancer. Annually, approximately 1,320 women receive a diagnosis of cervical cancer, with 744 dying due to the disease. It ranks as the ninth most common cancer among women aged 45 to 50 years [2].

Opportunistic screening using Pap testing takes place at healthcare facilities for in or outpatient Egyptian women aged between 20 and 50 years who attend for other gynecological problems primarily through universities and teaching hospitals [16].

Free screening services were provided to low-income women by the Egyptian Society for Colposcopy and Cervical Pathology which aims to train doctors and educate the public. A government-backed campaign “Journey of Thousand Kilometers” was launched in August 2023, visited 11 cities culminating along the Nile and went beyond education, with limited development of healthcare capacity to offer freely accessible screening and treatment services and set up twenty cervical cancer clinics across Egypt [17]. Regretfully, at present, national cervical screening practices and recommendations aren’t implemented in Egypt, neither the HPV vaccination nor cytological screening programs [2].

There is a lack of information about the exact situation of the HPV burden in the general population of Egypt. However, in the Northern Africa region, about 3.0% of women are infected with cervical HPV-16/18 sub-strain infection, which contributes to 78.9% of invasive cervical disorders [18]. The HPV vaccine became available in Egypt in 2009, it is not involved in the vaccination schedule, it costs about 30 $ (1500 Egyptian pounds), rendering it unaffordable for economically disadvantaged women [17].

Awareness of disease is essential for the successful implementation of screening programs, and healthcare professionals should possess adequate knowledge, efficacious insights, and effective strategies against cervical cancer to educate their communities [16]. The present study aimed to identify the predictors of cervical cancer screening, including the knowledge level, attitudes, beliefs, and preventive practices of female health professionals regarding cervical cell dysplasia. Furthermore, to address the potential obstacles associated with conducting cytological screening tests.

## Subjects and methods

### Study design and settings

This institutional-based cross-sectional study was carried out at two tertiary hospitals; Tanta and Menoufia University Teaching Hospitals (TUH and MUH) from January to March 2024. Tanta Teaching Hospital is located in the Gharbia governorate, comprising 23 clinical departments with a capacity of 1465 beds. Menoufia University Hospital is located in the city of Shebin El-Kom, Menoufia governorate, and has 22 clinical departments with a capacity of 600 beds. Both hospitals are multidisciplinary tertiary healthcare facilities, they include outpatient dental care units and they provide distinguished inpatients and outpatient medical care for about 8 million population residing in the Middle Nile Delta region of Egypt [19, 20]. Currently, there is no national screening program for cervical dysplasia. However, some local initiatives provide this screening tool at the Women’s Health outpatients clinic at TUH and MUH. Also, it is performed purposefully upon a special request by a gynecologist for suspicious patients.

### Target population and sampling

The target population was female health professionals working at TUH and MUH. Inclusion criteria enrolled the on-duty health practitioner females specifically; physicians, nurses, pharmacists, and dentists who had more than six months of experience.

Since tertiary health facilities don’t involve midwifery and the study targeted practitioner who potentially has direct contact with patients, we precluded laboratory technicians and midwives. The study also excluded interns, those who were on vacation during the period of the study, and practitioners who were suspected or confirmed to have cervical cell disorders.

Upon the information obtained from the employee affairs departments at both study settings, there were about 2780 and 2600 actively working healthcare provider women at TUH and MUH respectively during the period of the study, excluding technicians.

To ensure a more accurate sample representation, we have computed the sample size using the single population proportion formula; considering a 95% confidence interval (CI), 5% margin of error, and population proportion for knowledge, attitudes, and preventive practice were of 43%, 30%, and 2.2% respectively [10, 13]. Correspondingly, the total sample size was 384, 316, and 175 respectively. Therefore, after adding the 10% of non-response rate the overall sample size should be *n* = 963. After omitting invalid surveys due to incomplete or missing data the final recruited sample reached 1300 participants.

The participants were recruited using a multistage stratified random sampling technique. In the first stage, we randomly chose 15 clinical departments in each hospital including the pharmacy and the dentistry clinic, then the females working staff who met the inclusion criteria in each department were listed and categorized according to job ranks into (junior-mid-senior- senior). In the second stage, a random sample of 15–20 health professional from each job rank were taken, using a Microsoft Excel random numbering function.

A pilot study was held that included 25 health professional women to ensure the clarity and reasonability of the measuring tool’s content. The respondents’ feedback was taken, and then a few modifications were made accordingly. Results from the pilot study were incorporated into the final analysis.

## Data collection and measuring tool

Based on similar studies, an anonymous self-administered questionnaire was developed by the researchers [5, 13, 21], and attached as a supplementary file. The questionnaire was tested for its validity by three experts in gynecology, women’s health, and community medicine professionals, whose feedback was considered and modifications were made accordingly. The test-retest reliability was good at 85%, with internal consistency -Cronbach-alpha > 87%.

Participants were interviewed personally, and an electronic survey link was developed using Google survey forms to facilitate the process of data collection and entry. The questionnaire has a front page briefly describing the nature of the study and explaining its objectives. As well as a declaration of confidentiality and privacy of collected data, then the respondents were asked to provide formal consent to participate in the study.

The questionnaire comprised 4 sections; the 1 st included the sociodemographic data, medical history, and family history of the disease. The 2nd section assessed the knowledge of participants through 12 questions related to risk factors and screening tests of cervical cell disorders, the responses for each question were 1 for correct answer and 0 for incorrect and I don’t know the response. Then, the 3rd section included 7 items measuring the women ‘s attitudes towards cervical dysplasia; followed by a Likert scale from 1 strongly disagree to 5 strongly agree, the responses were summarized to agree, neither agree/disagree, and disagree for better data presentation. The questionnaire also includes 7 questions concerning insights about pap-smear screening with yes and no responses. The last section assessed the preventive practice against the disease including HPV vaccination and utilization of the implemented screening programs through 6 questions, as well as the potential barriers opposed to their periodic screening which were grouped into social, psychological, and health system factors.

Overall knowledge was summed up (maximum score 12) and categorized into good by achieving > 75%of total scores (> 9); fair at a range between 50 and 75% (6–9), and poor when attaining less than 50% of total scores (≤ 5). Likely, the attitude scale was computed with maximum score 35, and classified as positive > 26; neutral 17–26, and negative ≤ 16.

### Statistical analysis

The data manipulation process was executed using the Statistical Package of Social Sciences (SPSS) for Microsoft Windows version 26. Independent variables included in the sociodemographic data were presented in tables as frequency and percentages. The outcome variables were knowledge, attitudes, and preventive practices regarding cervical dysplasia and its screening. The proportion of correct knowledge, positive attitudes, and adequate preventive practice were compared across the 4 major specialties of health professionals using Chi Square test X^2^. The overall score of the three pillars was summed up and examined by the multivariate regression analysis to identify the likelihood of positive outcomes in relatedness to the independent determinants. The level of significance in the present study was adopted at *a p-value* < 0.05.

### Ethical consideration

The present study obtained the institutional review board (IRB) approval from the Research Ethics Committee at the Faculty of Medicine, Tanta University with approval reference no. 36264PR257/7/23. All participating health professional women provided approvals for the attached formed consent at the beginning of the Google form, subsequently after explaining the objectives of the study. Participants didn’t receive any incentives or financial/materials, they freely volunteered in the study. Anonymity and confidentiality were ensured for all respondents. The study strictly abided by the Helinski Declaration Ethical Principles 1964.

## Results

Table 1 shows the sociodemographic characteristics of the studied female health professionals whose number was 1300. Their mean age was 35.8 years, and more than three-quarters (80.9%) were from urban areas, with 76.3% being married. The mean age at marriage time was 25.3 years. 67.7% had 1–3 children, and 61.8% had 1–3 pregnancies. In regards to the job, they were divided into 4 groups: group I: 28.9% were physicians, group II 23.1% were nurses, group III 24.9% were pharmacists, and group IV 23.1% were dentists. Nearly two thirds of the respondents (62.5%) attained a postgraduate education level. The majority did not have a positive family history of cervical dysplasia nor received further education or training about cancer cytological screening after graduating (96.6%, and 85.2% respectively).

**Table 1:** Sociodemographic characteristics of the studied female health professionals (*n*=1300)

**Sociodemographic data**	**n (%)**
**Age (** **) **	
Mean SD	35.8±8.5
Range	24-58
**Years of Experience** ** (years)**	
Mean SD	11.6±3.5
Range	(1- 24)
**Residence:**	
Urban	1052(80.9%)
Rural	248(19.1%)
**Marital status:**	
Single	276(21.2%)
Married	992(76.3%)
Divorced	28(2.2%)
Widow	4(0.3%)
**Age at marriage (years)**	25.3±2.3
**Number of children:**	
0	312(24%)
1-3	880(67.7%)
>3	108(8.3%)
**Number of ** **:**	
0	312(24%)
1-3	803(61.8%)
4-5	173(13.35)
>5	12(0.9%)
**Job:**	
Physician	376(28.9%)
Nurse	300(23.1%)
Pharmacist	324(24.9%)
Dentist	300(23.1%)
**Education:**	
High school or diploma	100(7.7%)
Bachelor	383(29.5%)
Postgraduate	817(62.8%)
**Smoking status**	
Non -Smoker	1216(93.5%)
Smoker	84(6.4%)
**Income level:**	
Not enough	356(27.3%)
Just enough	526(40.5%)
More than enough	418(32%)
**Family history of cancer cervix (1st or 2nd degree relatives)**	
Yes	44(3.4%)
No	1256(96.6%)
**Did you receive any education or training about cancer cervix screening after graduation?**	
Yes	192(14.8%)
No	1108(85.2%)
**Did you recommend cancer cervix screening for any your patients before?**	
Yes	252(19.4%)
No	1048(80.6%)

Table (2) illustrates the level of knowledge regarding cervical cancer among the participants of the research. Regarding risk factors, 86.2% of the participants in Group I and 85.7% in Group II knew well that having several sexual partners was a risk factor. 47.9% in group I and 40% in group II thought that early sexual intercourse was a risk factor. The majority in groups I, II, and IV believed that infection with HPV and HIV increases the risk. Three-quarters of group III reported that cigarette smoking increases the risk of CC. Most of the respondents in the four groups knew that the use of IUD and oral contraceptive pills had no risk and approved that having a positive family history of CC raises the likelihood of acquiring CC.

**Table 2:** Comparison of good adequate knowledge regarding cervical dysplasia across the studied female health professiona (n=1300)Comparison of good adequate knowledge regarding cervical dysplasia across the studied female health professional (*n*=1300)

**Knowledge about cervical dysplasia**	**Group I (** ***n=*** **376)**	**Group II (** ***n=*** **300)**	**Group III (324)**	**Group IV (** ***n=*** **300)**	***p-*** **value** ^a^
**Risk factors:**					
Multiple sexual partners	324(86.2%)	260(86.7%)	121(37.3%)	168(56%)	0.0001*
Early sexual intercourse	180(47.9%)	120(40%)	20(6.2%)	60(20%)	0.0001*
HPV infection (human papillomavirus)	340(90.4%)	260(86.7%)	101(31.2%)	216(72%)	0.0001*
Infection with the human immunodeficiency virus (HIV AIDS)	268(71.3%)	240(80%)	121(37.3%)	192(64%)	0.0001*
Cigarette smoking	160(42.6%)	140(46.7%)	244(75.3%)	96(32%)	0.0001*
Use of contraceptive, Intrauterine device (Loop)	80(21.3%)	100(33.3%)	143(44.1%)	48(16%)	0.0001*
Positive family history	308(81.9%)	240(80%)	284(87.7%)	228(76%)	0.0001*
Intake of Oral contraceptive pills	116(30.9%)	140(46.7%)	124(38.3%)	96(32%)	0.0001*
**Methods of screening:**					
Pap smear	332(88.3%)	200(66.7%)	224(69.1%)	156(52%)	0.0001*
Visual inspection of cervix	176(46.8%)	180(60%)	144(44.4%)	84(28%)	0.0001*
Human papillomavirus DNA testing	228(60.6%)	200(66.7%)	41(12.7%)	144(48%)	0.0001*
Liquid-based cytology	148(39.4%)	200(66.7%)	120(37%)	84(28%)	0.0001*
**Total Knowledge level **					
Poor	52(13.8%)	60(20%)	120(37%)	120(40%)	0.0001*
Fair	208(55.3%)	100(33.3%)	183(56.5%)	120(40%)	
Good	116(30.9%)	140(46.7%)	21(6.5%)	60(230%)	

Group I: physicians, Group II: Nurse, Group III: Pharmacist, Group IV: Dentist. ^a^*X*^2^test, * significance

Regarding the methods of CC screening, 88.3% of females in group I, 66.7% in group II, and 69.1% in group III were adequately aware of pap smear as a golden screening method. In group II of nurses: 60%, 66.7%, and 66.7% respectively believed that visual inspection of the cervix, human papillomavirus DNA testing, and liquid-based cytology are valid methods of screening.

**Fig. 1:**
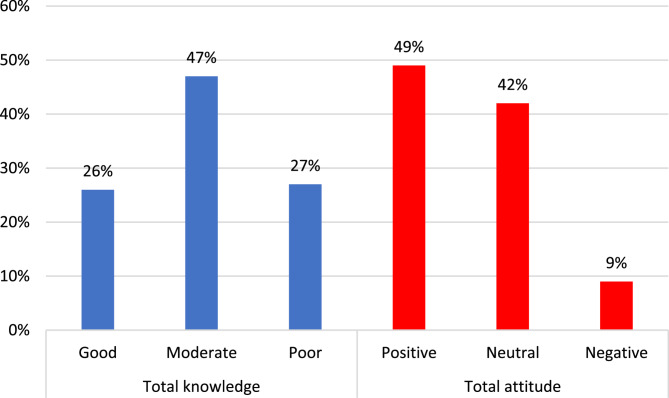
Level of knowledge and attitude across the studied health professional women regarding cervical dysplasia and screening

Figure (1) illustrates levels of knowledge and attitude across the studied female health professionals regarding cervical dysplasia and screening, where 47% of them had moderate knowledge and 49.2% had a positive attitude regarding cervical cell dysplastic disorders as well as its screening.

Concerning the overall level of knowledge, only 25% of health professional participants had a good level of knowledge (Fig. 1) and within the four groups, more than half of Group I and Group III possessed a fair level of knowledge. Additionally, 46.7% of Group II presented a good level of knowledge, but 40% of Group IV had poor and fair knowledge as shown in Table 3.

Table (3) discusses the participants’ attitudes and beliefs towards CC. Among the studied women in group II, 60% agreed that CC is highly prevalent and is a leading cause of death among all malignancies. In group IV, 64% of participants agreed that any young woman can acquire CC. 68.1% of women in group I believed that cancer cervix is not transmitted. Most of the participants accepted that screening is beneficial in the prevention of cancer cervix. In addition, 66% in Group I, 86.7% in Group II, 75.3% in Group III, and 68% in Group IV believed that cervical cancer cervix is a curse. The total attitude was summed up and it was found that 8.9% had negative attitudes and 49.2% had a positive attitude towards cervical dysplasia (Fig. 1). We revealed that 51.1% of females in Group I showed a fair attitude, but 53% in Group II, 56.8% in Group III, and 48% in Group IV showed a good attitude.

**Table 3:** Attitudes regarding cervical dysplasia of the studied female health professionals (*n*=1300)

**Participants’ attitudes towards cervical dysplasia **	**Group I** **(** ***n*** **=376)**	**Group II** **(** ***n*** **=300)**	**Group III** **(324)**	**Group IV** **(** ***n*** **=300)**	***p-*** **value **
Cervix is highly prevalent and is a leading cause of deaths amongst all malignancies	152(40.4%)	180(60%)	143(44.1%)	132(44%)	0.0001*
Any young woman can acquire cancer cervix	204(54.3%)	180(60%)	184(56.8%)	192(64%)	0.0001*
Cancer cervix cannot be transmitted	256(68.1%)	140(46.7%)	141(43.5%)	156(52%)	0.0001*
Screening helps in prevention of cancer cervix	332(88.3%)	260(86.7%)	284(87.7%)	240(80%)	0.0001*
Cancer cervix is a curse (very bad something)	248(66%)	260(86.7%)	244(75.3%)	204(68%)	0.0001*
**Total attitude:**					0.0001*
Negative	32(8.5%)	0(0%)	60(18.5%)	24(8%)	
Neutral	192(51.1%)	141(47%)	80(24.7%)	132(44%)	
Positive	152(40.4%)	159(53%)	184(56.8%)	144(48%)	

Group I: physicians, Group II: Nurse, Group III: Pharmacist, Group IV: Dentist. ^a^Chi *X*^2^test, * significance

Table (4): Attitudes, beliefs and Knowledge of pap smear tests across the studied female health professionals(*n* = 1300). Table 4 assesses the knowledge and practice of pap smears among the study groups. 75.5% of respondents in Group I, 60% in Group II, and 68% in Group IV disagreed that screening was harmful to the client. 48.9% of females in Group I disagreed that screening of cc is expensive whereas 46.7% in Group II agreed. Among the participants, 35.1% in Group I and 44.1% in Group III demonstrated that the appropriate age for screening is 30 years. However, 80% of participants in group II and 72% in group IV are unaware of the proper age for screening.

**Table 4:** Attitudes, beliefs and Knowledge of pap smear test across the studied female health professionals (*n*=1300)

**Assessing beliefs regarding pap smear screening test **	**Group I** **(** ***n*** **=376)**	**Group II** **(** ***n*** **=300)**	**Group III** **(324)**	**Group IV** **(** ***n*** **=300)**	***p-*** **value **
**Screening causes harm to the **					0.0001*
Agree	40(10.6%)	80(26.7%)	40(12.3%)	24(8%)	
Disagree	284(75.5%)	180(60%)	120(37%)	204(68%)	
Neither agree nor disagree	52(13.8%)	40(13.3%)	164(50.6%)	72(24%)	
**screening for cervical cancer is **					0.0001*
Agree	76(20.2%)	140(46.7%)	81(25%)	36(12%)	
Disagree	184(48.9%)	80(26.7%)	60(18.5%)	84(28%)	
Neither agree nor disagree	116(30.9%)	80(26.7%)	183(56.5%)	180(60%)	
**What is the proper age at which Pap smear test ** ** be started?**					0.0001*
From puberty	36(9.6%)	20(6.7%)	40(12.3%)	12(4%)	
From 20 years	84(22.3%)	40(13.3%)	20(6.2%)	24(8%)	
From 30 years	132(35.1%)	0(0%)	143(44.1%)	36(12%)	
After menopause	32(8.5%)	0(0%)	20(6.2%)	12(4%)	
I don’t know	92(24.5%)	240(80%)	101(31.2%)	216(72%)	
**Best time for doing Pap smear test:**					0.0001*
During menstrual	0(0%)	0(0%)	40(12.3%)	0(0%)	
A week after period	116(30.9%)	60(20%)	40(12.3%)	60(20%)	
Not sure	260(69.1%)	240(80%)	244(75.3%)	240(80%)	
**Pap smear test should be done by** **:**					0.0001*
Any physician	4(1.1%)	0(0%)	0(0%)	0(0%)	
Gynecologist	248(66%)	160(53.3%)	224(69.1%)	180(60%)	
The 1^st^ two	56(14.9%)	20(6.7%)	20(6.9%)	12(4%)	
Trained nurse	8(2.1%)	20(6.7%)	0(0%)	12(4%)	
Not sure	60(16%)	100(33.3%)	80(24.7%)	96(32%)	
**What is the proper interval for doing Pap smear test?**					0.0001*
Monthly	0(0%)	0(0%)	0(0%)	12(4.2%)	
Yearly	204(54.3%)	120(40%)	163(50.3%)	60(20.8%)	
After menopause	24(6.4%)	0(0%)	20(6.2%)	0(0%)	
Not sure	148(39.4%)	180(60%)	141(43.5%)	216(75%)	
**If there is abnormality in Pap smear test results, what should be done** **?**					0.0001*
Leave it to God and	8(2.1%)	0(0%)	0(0%)	0(0%)	
Do confirmatory lab tests	280(74.5%)	120(40%)	264(81.5%)	228(76%)	
Not sure	88(23.4%)	180(60%)	60(18.5%)	72(24%)	
**What are the benefits of Pap smear ** **?**					0.0001*
Detection of any early abnormal changes in the cervix	56(14.9%)	40(13.3%)	20(6.2%)	72(24%)	
Early detection of cervical cancer	52(13.8%)	60(20%)	41(12.7%)	36(12%)	
The 1^st^ two items	224(59.6%)	80(26.7%)	183(56.5%)	132(44%)	
Not sure	44(11.7%)	120(40%)	80(24.7%)	60(20%)	
**Pap smear test is done using:**					0.0001*
Transvaginal ultrasound	32(8.5%)	60(20%)	0(0%)	12(4%)	
Vaginal brushing	200(53.2%)	60(20%)	123(38%)	84(28%)	
Not sure	112(29.8%)	160(53.3%)	181(55.9%)	204(68%)	
Others	32(8.5%)	20(6.7%)	20(6.2%)	0(0%)	

Group I: physicians, Group II: Nurse, Group III: Pharmacist, Group IV: Dentist. ^a^*X*^2^test, * significance

Most of the women in the four groups (69.1%, 80%, 75.3%, and 80% respectively) were uncertain about what is the best time for undergoing a Pap smear test. Over 50% of participants in the four groups agreed that a gynecologist is the one who should perform a Pap smear. In groups I and III (54.3% and 50.3% respectively) respondents were aware of the proper interval for a pap smear which is once a year, while 60% in group II and 75% in group IV were unaware. Around 75% of women in groups I, II, and IV would undergo confirmatory testing in the case of detecting any abnormalities in their pap smear results. However, it is unclear for 60% of women in group II what measures they have to take in such conditions. The majority of women in groups I, III, and IV (59.6%, 56.5%, and 44% respectively) were knowledgeable about the benefits of pap smear. Among the respondents in group I, 53.2% had enough knowledge about the procedure for conducting a pap smear, however, most of the participants in the other three groups were unsure.

Tables 5 and 6 summarizes the preventative actions for cervical cancer among the studied females. A high proportion of responders in all groups had not been vaccinated against HPV (97.7%, 100%,100%, and 100% respectively) or had undergone a pap smear (89.4%, 93.3%, 100%, and 100% respectively).

**Table 5:** Preventive practices against cervical dysplasia of the studied female health professionals (*n*=1300)

**Preventive practices**	**Group I** **(*n*=376)**	**Group II** **(*n*=300)**	**Group III** **(324)**	**Group IV** **(*n*=300)**	***p-*value **
**Have you ever vaccinated against human papillomavirus?**					
No	368(97.9%)	300(100%)	324(100%)	300(100%)	0.001*
Yes	8(2.1%)	0	0	0	
**Have you undergone pap smear test?**					
No	336(89.4%)	280(93.3%)	324(100%)	300(100%)	0.0001*
Yes	40(10.6%)	20(6.7%)	0(0%)	0(0%)	
**If yes; what were the test results?**					
Normal finding	40(10.6%)	20(6.7%)	0(0%)	0(0%)	0.0001*
Not done	336(89.4%)	280(93.3%)	324(100%)	300(100%)	
**If yes; when was the last scan time?**					
One year ago	20(5.3%)	0(0%)	0(0%)	0(0%)	0.0001*
≥ two years ago	20(5.3%)	20(6.7%)	0(0%)	0(0%)	
Not done	336(89.4%)	280(93.3%)	324(100%)	300(100%)	
**What are the reasons for doing the tests?**					
General health checks up	12(3.2%)	8(2.7%)	0(0%)	0(0%)	0.0001*
Doctor recommendation	8(2.1%)	5(1.7%)	0(0%)	0(0%)	
Complain from itching, bleeding, discharge, etc	20(5.3%)	7(2.3%)	0(0%)	0(0%)	
Not done	336(89.4%)	280(93.3%)	324(100%)	300(100%)	

Group I: physicians, Group II: Nurse, Group III: Pharmacist, Group IV: Dentist * significance

**Table 6:** Potential barriers opposing screening of cervix cell disorders among the studied female health professionals (*n*=1300)

**Barriers against CC screening**	**Group I** **(*n*=376)**	**Group II** **(*n*=300)**	**Group III** **(324)**	**Group IV** **(*n*=300)**	***p-*value**
**Psychological:**					
* The culture norms*					
Yes	244(64.9%)	200(66.7%)	100(30.9%)	144(48%)	0.0001*
No	132(35.1%)	100(33.3%)	224(69.1%)	156(52%)	
* No complaint*					
Yes	300(79.8%)	240(80%)	181(55.9%)	228(76%)	0.0001*
No	76(20.2%)	60(20%)	143(44.1%)	72(24%)	
* Screening procedure is painful *					
Yes	144(38.3%)	200(66.7%)	101(31.2%)	132(44%)	0.0001*
No	232(61.7%)	100(33.3%)	223(68.8%)	168(56%)	
* Screening is embarrassing and shame *					
Yes	184(48.9%)	140(46.7%)	40(12.3%)	72(24%)	0.0001*
No	192(51.1%)	160(53.3%)	284(87.7%)	228(76%)	
* Screening is not helpful to prevent God willing *					
Yes	108(28.7%)	60(20%)	40(12.3%)	24(8%)	0.0001*
No	268(71.3%)	240(80%)	284(87.7%)	276(92%)	
* Fear of positive finding*					
Yes	220(58.5%)	180(60%)	140(43.2%)	144(48%)	0.0001*
No	156(41.5%)	120(40%)	184(56.8%)	156(52%)	
**Socioeconomic:**					
* Screening test is expensive*					0.0001*
Yes	72(19.1%)	80(26.7%)	101(31.2%)	72(24%)	
No	224(59.6%)	20(6.7%)	143(44.1%)	180(60%)	
I don’t know	80(21.3%)	200(66.7%)	80(24.7%)	48(16%)	
* I don’t have enough time for screening*					0.0001*
Yes	204(54.3%)	160(53.3%)	61(18.8%)	144(48%)	
No	156(41.5%)	60(20%)	203(62.7%)	156(52%)	
I don’t know	16(4.3%)	80(26.7%)	60(18.5%)	0(0%)	
* Screening test isn’t available in my workplace*					0.0001*
Yes	200(53.2%)	180(60%)	163(50.3%)	216(72%)	
No	132(35.1%)	20(6.7%)	121(37.3%)	72(24%)	
I don’t know	44(11.7%)	100(33.3%)	40(12.3%)	12(4%)	
* My husband might not agree to do screening*					0.0001*
Yes	88(23.4%)	100(33.3%)	0(0%)	36(12%)	
No	252(67%)	120(40%)	304(93.8%)	252(84%)	
I don’t know	36(9.6%)	80(26.7%)	20(6.2%)	12(4%)	
**Healthcare system barriers:**					
* There is long waiting time at health facility to do screening *					
Yes	184(48.9%)	200(66.7%)	80(24.7%)	96(32%)	0.0001*
No	192(51.1%)	100(33.3%)	244(75.3%)	204(68%)	
* I don’t feel comfortable with male physician who offers to screen*					
Yes	276(73.4%)	180(60%)	201(62%)	216(72%)	0.0001*
No	100(26.6%)	120(40%)	123(38%)	84(28%)	
* I don’t know which health facility offering cancer cervix screening *					
Yes	240(63.8%)	240(80%)	223(68.8%)	240(80%)	0.0001*
No	136(36.2%)	60(20%)	101(31.2%)	60(20%)	
* I do not know whom to consult for undergoing this test*					
Yes	164(43.6%)	200(66.7%)	100(30.9%)	168(56%)	0.0001*
No	212(56.4%)	100(33.3%)	224(69.1%)	132(44%)	

Group I: physicians, Group II: Nurse, Group III: Pharmacist, Group IV: Dentist. ^a^Chi *X*^2^test, * significance

Table (7) analyzes the independent sociodemographic about the knowledge, and attitude of the studied health professional women. Concerning knowledge, the predictor variables age and urban residency had statistically significant negative coefficients. The age coefficient is represented by the value of beta, which was − 0.04, s.e = 0.01, and the *p* is 0.0001. The urban residence coefficient was − 0.6, s.e = 0.1, *p* = 0.0001. This suggests that increasing the participants’ age and living in urban areas were associated with low levels of knowledge. Meanwhile, being employed as a physician and nurse, possessing a higher level of education, many years of experience, and having a positive family history of cervical cancer contributed to increased knowledge levels.

**Table 7:** Multivariate regression analysis of knowledge and attitudes outcomes of the studied female health professional (*n*=1300)

Independent sociodemographic	knowledge					Attitude				
	B	s.e	95%Confidence interval		*p*	B	s.e	95%Confidence interval		*p*
			lower	Upper				Lower	Upper	
Age	−0.04	0.01	−0.06	−0.03	0.000*	−0.08	0.02	−0.1	−0.05	0.000*
**Residence**										
Urban	−0.6	0.1	−0.9	−0.4	0.000*	−0.9	0.1	−1.2	−0.6	0.000*
Rural ^a^										
**Marital status**										
Not married	0.2	0.1	−0.03	0.5	0.09	0.01	0.1	−0.2	0.3	0.9
Married ^a^										
**Job**										
Physician	1.02	0.1	0.7	1.3	0.000*	−0.2	0.2	−0.5	0.1	0.3
Nurse	1.4	0.2	1.1	1.7	0.000*	0.5	0.2	0.2	0.8	0.004*
Pharmacist	−0.2	0.2	1.1	1.7	0.2	0.4	0.2	0.1	0.7	0.02*
Dentist ^a^										
**Education**										
High school or diploma	−0.6	0.2	−0.9	−0.2	0.002*	−1.1	0.2	−1.5	−0.7	0.000*
Bachelor	−0.7	0.1	−0.9	−0.4	0.000*	−0.3	0.1	−0.6	−0.04	0.02*
Postgraduate ^a^										
**Experience**	−0.02	0.007	−0.03	−0.008	0.001*	0.001	0.007	−0.01	0.01	0.9
**Family history**										
-ve history	−1.2	0.3	−1.7	−0.6	0.000*	−0.2	0.3	−0.8	0.4	0.5
+ve history ^a^										

*Significance *p*<0.05

^a^reference group

As for attitude, the negative coefficients for the predictor variables age and urban residency (beta=−0.08, −0.9 respectively) were significant (*p* = 0.0001). This suggests that when participants’ age increases, urban residency is associated with a negative attitude. On the other hand, having a high level of education, and being a nurse and pharmacist is linked to a positive attitude.

**Fig. 2:**
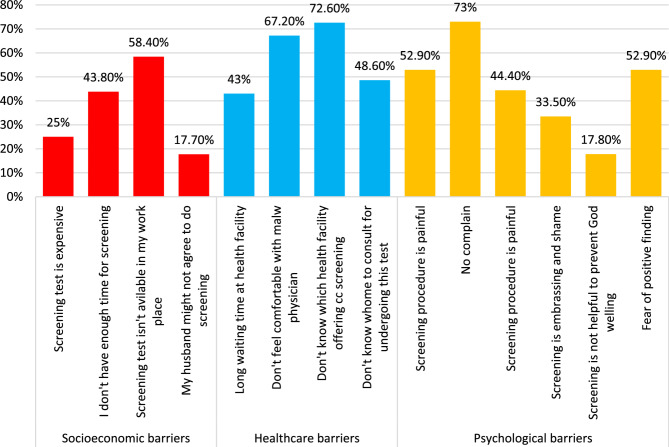
Barriers to screening of cervical dysplasia among the studied female health professionals

Figure (2) depicts the potential barriers against CC screening which encompass socioeconomic, healthcare system, and psychological obstacles; where 58.4% of women reported that the screening tests aren’t available at their workplace, 72.6% reported that they don’t know which health facility could provide CC screening and 73% of women who didn’t perform screening test so far because they don’t have any complains.

## Discussion

Gynecological malignancies in women are a major public health concern since they are common and often fatal diseases. Cancer cervix (CC) is the second most common cancer in women globally, following breast cancer, and ranks first among gynecologic cancers. Globally, about 266,000 women die annually from CC, with 87% of these occurring in less developed areas [22].

Since cervical dysplasia is a preventable disease with early detection, a key approach to prevention is to increase public awareness and ensure that healthcare personnel possess accurate knowledge regarding prevention and screening techniques [23]. The National Cervical Cancer Coalition (NCCC), a nonprofit organization dedicated to the prevention of cervical cancer and HPV disease in collaboration with WHO, has asserted that it is essential for all healthcare workers to offer counseling and health education on CC prevention to females, it also emphasizes on enhancing the knowledge of healthcare practitioners to educate people about CC prevention, screening, and early detection, and contribute to effective struggle against CC. Upon reviewing the literature, it is evident that healthcare providers across the world possess a limited degree of knowledge regarding CC early diagnosis, prevention, and screening [4, 5, 10, 21].

Of 1300 study participants, only 14.8% received further education or training about cancer cervix screening after graduation. The matter that implicates the observed limited information and infrequent updates contributes to the low acceptance of screening.

Our figures are consistent with a study conducted by Altunkurek S., (2022) in Mogadishu, the capital of Somalia, it studied the knowledge and attitudes of healthcare providers, who found that 22.1% of the respondents declared that they got training on CC as a part of their vocational training. Also, noted that 16.8% of the participants received in-service training after graduation, and the remaining said that they did not receive any training [23]. Similarly, the current study findings are consistent with the study conducted by Mwalwanda A., et al. (2024) in Indonesia, which reported that 84.3% of participants didn’t have CC screening education as a component of the facility program [24].

Findings were replicated by Habas et al. 2025 who demonstrated, that most of participated women didn't receive any education on cervical cancer throughout their life [25].

Concerning risk factors of cervical dysplasia, the present study elucidated that the majority in all groups agreed that having several sexual partners, early sexual intercourse, infection with HPV and HIV, cigarette smoking, and positive family history of CC were potential risk mediators. This finding aligned with Majid E et al., (2022) study in Karachi, Pakistan, which revealed that the frequently observed risk factors were multiple sexual partners (73.2%), initiating sexual intercourse at a young age (46%), smoking (37.8%), foul-smelling discharges (63.7%), and post-coital bleeding (66.6%) [26]. Similarly, a study conducted by Mwalwanda A et al., (2024) revealed that 18.6% and 15.7% of study participants respectively identified multiple partners, and early sexual debut as risk factors [24].

The study identified a notable good knowledge of participants regarding the methods of cervical cell disorders screening, as more than half of study participants in all professions were aware of Pap smear as a screening method, believed that visual inspection of the cervix, human papillomavirus DNA testing, and liquid-based cytology are methods of screening. On the same line, Dulla D, et al., (2017) in Ethiopia declared that (77.1%) of responders were aware of methods used to detect premalignant cervical lesions, and (37.6%) of them identified visual inspection with acetic acid as a screening approach [27].

Concerning the total level of knowledge within the four groups, the major proportion in groups I, III, and IV(55.3%, 56.5%, and 40% respectively) possessed a fair level of knowledge. 46.7% of Group II had a good knowledge. The observed discrepancy could be attributed to variations in the scientific backgrounds across the four professional groups.

The consistent finding was reported by Abebaw, E. (2022) in Northwest Ethiopia and highlighted that 44.3% of the respondents knew the risk factors related to cervical cancer, while 43.8% of females demonstrated sufficient knowledge regarding cervical cancer screening [28].

Moreover, Chawla B., (2021) who conducted a systematic review of a total of 22 studies and included 6811 health professionals in India, polled that the general knowledge of CC among health professionals was 75.1% and the awareness level of risk factors was considered sufficient [13]. Likely, Obol JH et al., (2021) who conducted a study on healthcare professionals in Uganda observed that 60% of the participants showed sufficient knowledge concerning cervical cancer [29].

Notably, a similar study was held between Egyptian working women, revealed that 76% of respondents had poor knowledge level regarding cancer cervix, the majority 81.2% expressed negative attitudes towards cervical disorders and early detection screening and only 5.3% performed Pap smear test [14].

In contrary with our figures, a similar study conducted by Heena H, (2019) in Saudi Arabia displayed that only 4% of responders had good knowledge [30]. Another study conducted by Ampofo et al. (2020) revealed that nearly 82% of women in Kenyan, Southern Ghana, have poor knowledge regarding cancer cervix [31]. The discrepancies between studies may be due to differences in personal beliefs, information, policy, time, training opportunities for the care providers, and variability in healthcare policies within the study areas.

Concerning the participants’ attitudes about cervical dysplasia, 60% of the women in group II stated that it is highly common and a primary cause of mortality among all types of cancer. In group IV, 64% of respondents declared that any young woman has a chance to obtain CC. In group I, 68.1% of females accepted the belief that cervical cancer is not transmitted. Meanwhile, the majority of groups agreed that screening is useful in preventing cervical cancer. Interestingly, 66%, 86.7%, 75.3%, and 68% of participants in Groups I, II, III, and IV respectively believed that CC is a curse. This adverse belief could contribute to the abstaining of practitioners to advise their patients to undergo screening in 80.6%, as well as explain why the majority of our respondents didn’t perform screening nor receive the HPV vaccine.

The present study findings are consistent with a study conducted in Indonesia, presented that the majority of the female health workers were aware of CC (100%) approximately 97.1% recognized that CC is preventable and 75.7% identified HPV as the underlying cause [24].

The total attitude was observed to be positive and neutral across the four groups. These findings are aligned with the metanalysis study of Chawla B., (2021) who declared a positive attitude about screening in a large proportion of health workers, and 85.47% of participants owned a positive attitude regarding screening [13]. Likely, a study conducted by Daniyan BC et al. (2019) in Nigeria examined the attitude of FHWs toward cervical cancer screening and reported a favorable attitude toward CC screening among FHWs [32].

Regarding study groups’ knowledge and practice of Pap smear; about 46.3% in Group II agreed that screening for CC is expensive. Regretfully, the majority were unaware of the appropriate age for screening and uncertain about what is the best time for undergoing a Pap smear test. While, more than half of the participants in the four groups agreed that a gynecologist is the one who performs a Pap smear, and was knowledgeable about the benefits of a Pap smear. However, the majority of participants in the other three groups excluding physicians were unsure of the pap smear procedure.

Our figures were supported by Obol JH et al., (2021) who stated that most of the participants were aware of the significance of early screening for CC in its prevention, whereas only 16% correctly identified the appropriate age group for cervical cancer screening [29].

The study deduced that the female health workers’ knowledge about the recommended CC screening interval and target population eligible for screening was as poor as documented by two studies held in Nigeria, which stated that pap smears were reported as the most popular screening method and the stratified CC screening knowledge by a cadre of female health care workers, was observed to be ‘profession-dependent’ as doctors were more knowledgeable compared to others [33, 34].

Regarding the preventive practice for cervical dysplasia, most of the professionals had not administered the HPV vaccine (99.5%) nor undergone a pap smear (95.6%). Our observations are replicated in several studies worldwide, which reported similarly low levels of screening among nurses and health workers in Southeast Asia and Africa with screening uptake rates of 16.6%, 15.0%, 18.9%, and 11.4% [27, 35–38]. In the same context, the study conducted by Eze et al. (2018) in Nigeria, found that just 22% of healthcare practitioners have done a Pap smear and only 28% of respondents who had performed a Pap smear said they had done it more than once. Even fewer healthcare practitioners (11%) had expertise in visual inspection of acetic acid (VIA), with 29% having conducted it more than ten times [38].

The low rate of screening acceptance may be attributed to the fear of receiving a false diagnosis, the discomfort experienced throughout the screening process, and being uncomfortable if investigated by male physicians.

The multivariate analysis of our study elaborated that age and urban residency were independent predictor variables of knowledge level, where the increased participant’s age and being from urban areas were associated with decreased knowledge. Employed as a physician and nurse, earning a high level of education, years of experience, and having a positive family history of cervical disorders, all significantly contributed to increased knowledge level. Likely, Eze et al., (2018) study found that the level of awareness regarding risk factors for CC was associated significantly with occupation, as 70% of doctors successfully identified all risk factors compared to 48% of medical students and nurses, and 36% of midwives [38].

Additionally, Al-Shamlan., (2023) who studied factors associated with the utilization of CC screening among healthcare professionals in Saudi Arabia demonstrated that CC screening was significantly common among professionals who attained a master’s degree or higher. Also, it was higher among HCWs who had a family history of CC. On the other hand, nurses, pharmacists, and technicians were significantly less likely than physicians to utilize cervical cancer screening [39].

Furtherly, our findings are supported by the study conducted by Abebaw E, et al. (2022) in Northwest Ethiopia who demonstrated that physicians had a 2.4 times higher likelihood (adjusted odds ratio = 2.4) of having a positive attitude towards cervical disorder screening compared to other medical professions. Similarly, they found that midwives had a 1.3 times higher likelihood (adjusted odds ratio = 1.3) of having a good attitude than other medical occupations [22].

A prior study involved gynecologists and obstetricians in Egypt reported that less than half of participants (45%) possessed poor-to-fair knowledge, while 57% showed negative-to-fair positive attitudes regrading screening measures and vaccination against HPV, and 44% had ever-done Pap smear and interestingly 45% of physicans had ever advised their patients to take the HPV vaccine [40].

A relevant study conducted by Aggarwal et al. (2023) found that the knowledge about CC screening through a Pap smear was even higher than among our study participants, who included both medical and paramedical staff (97%) and proved to be occupationally dependent [41].

The present study demonstrated barriers against CC screening; for socioeconomic barriers, nearly half of women (58.4%) reported that the screening test isn’t available in their workplace. Regarding healthcare barriers, 72.6% of respondents reported that they didn’t know the health facility that offers screening. For psychological barriers, nearly three-fourths of females (73%) didn’t perform the test as there was no complaint.

Parallelly, a similar study in Egypt reported that the most hindering obstacles for Pap smear testing; were fear of detecting early signs of neoplasia (23.9%) and 17.8% mentioned the test is embarrassing [14].

Previous literature demonstrated a multitude of obstacles to CC screening participation among HCWs including insufficient knowledge, expensive screening costs, fears about misdiagnosis, discomfort or pain felt during screening, varying levels of personal health involvement, gender of the screener, inadequate privacy during screening, and dissatisfaction with the provided health service [31, 40].

### Strengths and limitations

The cross-sectional design of this study, suggests a chance of recalling and social desire bias and interfering with the causality relationship. As well, the study was conducted at two health facilities that might hinder generalizability of results. The perception of barriers was based on self-reporting, making it challenging to assess the validity of these answers. On the other hand, the multicentric settings of the study allowed for involving a broad sector of health professional women. The questionnaire arouses the participants’ interest and eagerness to identify more about risk factors, clinical manifestation, and preventive practices against cervical dysplasia.

## Conclusion

Less than half of the participants possessed moderate knowledge and good insights toward CC and its screening. A significant percentage of respondents had not received HPV vaccination or undergone a Pap smear testing. Increasing the participants’ age and living in urban areas were associated with low levels of knowledge and negative attitudes. Meanwhile, attaining a higher level of education contributed to an increased knowledge level and positive attitudes. The main obstacles to CC screening were unavailability of the test at the workplace, inaccessibility to health facilities providing CC screening, and absence of physical complaint.

### Recommendations

Study findings advocate for the development of a nationwide campaign to be implemented at different levels of healthcare delivery. In addition, endorse the geographical coverage of the established initiative of “Journey of a Thousand Kilometers” to enhance undergoing cervical cancer screening. Education of women regarding risk factors could improve access to screening services. The study also emphasizes cancer vaccines to be incorporated into the standard immunization schedule as a cost-effective measure. Furthermore, provide complimentary cervical cancer screening clinics nationwide by the Egyptian Society for Colonoscopy and Cervical Pathology.

## Supplementary Information

Supplementary Material 1.

## Data Availability

Data generated and analayzed in this study, is available with corresponding author on a reasonable request.

## Abbreviations

CC: Cervical cancer
FHCWs: Female healthcare workers
IRB: Institutional Review Board
HCW: Health care worker
HPV: Human papilloma virus
hr-HPV: High risk human papilloma virus
TUH: Tanta University Hospitals
MUH: Menounfia University Hospitals
Pap: Panicolaou test
