# Supplementary material for: Preventive insights and practices of female health professionals regarding cervical cell dysplasia: a cross-sectional study in Egypt
Source: BMC Public Health. 2025 Nov 6;25:3809. doi: 10.1186/s12889-025-24004-4 (PMC12590728; doi:10.1186/s12889-025-24004-4)
Supplement: Supplementary file 1 — Supplementary Material 1. [file 12889_2025_24004_MOESM1_ESM.pdf]

**Preventive insights and practices of female health professionals regarding cervical cell dysplasia: an observational study in Egypt**

This study is conducted by researchers in Public Health and Community Medicine department, at Faculty of Medicine Tanta University, and Faculty of Nursing , Menofia University.

This research is related to women's health which aims to study the awareness level and insights of health professional females regarding cancer cervix risk factors, as well the preventive practice and associated barriers that might hinder you against cancer cervix screening.

We would be happy if you kindly participate in our upcoming research that could contribute to the quality of health services of Egyptian women.

We do affirm that the collected data will be used for scientific purposes only. This questionnaire is anonymous with no referring to your personal identity and takes about 10 mins to fulfill. Confidentiality and privacy are completely assured.

The investigators would be appreciated to your inputs and willing to expand further detailed information through this phone number : +2 01288055739 or Email : mera.ramadan@med.tanta.edu.eg, Doaam3y@gmail.com

---

## Consent Form

Dear participant ,

- All the study procedures strictly abided by the Helsinki Declaration Ethical Principles 1964. The present study obtained IRB approval from the Research Ethics Committee at the Faculty of Medicine, Tanta University with approval reference no. 36264PR257/7/23.
- There aren't any potential harms or threats for participation in the study.
- The participation is completely voluntary with no enforcement or incentives, considering that you can withdraw from the study at any time with no obligation.
- The research objectives include :
  - identify the predictors of cancer cervix screening; including the level of awareness, attitudes, insights of female health professionals against cervical cell dysplasia.
  - determine preventive practices conducted by female health professionals against cervical dysplasia
  - address the potential barriers against performing cytological screening tests.
- Are you willing to participate in the study ? ☐Yes ☐No

*If yes ; kindly respond promptly to the items in the questionnaire*

## Section I: Socio demographic data

- Age: ……. years
- Residence: ☐ urban ☐ rural
- Marital status: ☐single ☐married ☐divorced ☐ widow
- Age at marriage:
- Number of children: ☐0 ☐1-3 ☐ >3
- Number of pregnancies:
- Job: ☐physician ☐nurse ☐pharmacist ☐dentist
- Level of education: ☐High school or diploma. ☐Bachelor. ☐post graduate studies
- Is there a family history of cancer cervix (1st and 2nd degree relatives)? ☐Yes ☐No
- Did you receive any education or training about cancer cervix screening after graduation?
  ☐Yes ☐No
- Did you recommend cancer cervix screening for any your patients before? ☐Yes ☐No

**Section II: Knowledge and Insights about cervical dysplasia** *Kindly tick Yes or No responses according to your knowledge for the following items;*

| Items of the knowledge scale | Yes | No | I don't know |
|---|---|---|---|
| **Risk factors** | | | |
| o multiple sexual partners | ☐ | ☐ | ☐ |
| o early sexual intercourse | ☐ | ☐ | ☐ |
| o HPV infection (human papillomavirus) | ☐ | ☐ | ☐ |
| o infection with the human immunodeficiency | ☐ | ☐ | ☐ |
| virus | ☐ | ☐ | ☐ |
| o cigarette smoking | ☐ | ☐ | ☐ |
| o use of contraceptive intrauterine device (loop) | ☐ | ☐ | ☐ |
| o positive family history | ☐ | ☐ | ☐ |
| o intake of oral contraceptive pills | ☐ | ☐ | ☐ |
| **What are the methods of screening?** | | | |
| o Pap smear | ☐ | ☐ | ☐ |
| o visual inspection of cervix | ☐ | ☐ | ☐ |
| o human papillomavirus DNA testing | ☐ | ☐ | ☐ |
| o liquid-based cytology | ☐ | ☐ | ☐ |

**Section III: Attitudes and Insights about cancer cervix and PAP smear** *Kindly tick the appropriate response to the following items;*

| Items | Strongly disagree | Disagree | Neither agree nor disagree | Agree | Strongly agree |
|---|---|---|---|---|---|
| Cancer cervix is highly prevalent and is a leading cause of deaths amongst all malignancies | ☐ | ☐ | ☐ | ☐ | ☐ |
| Any young woman can acquire cancer cervix | ☐ | ☐ | ☐ | ☐ | ☐ |
| Cancer cervix cannot be transmitted | ☐ | ☐ | ☐ | ☐ | ☐ |
| Screening helps in prevention of cancer cervix | ☐ | ☐ | ☐ | ☐ | ☐ |

| | | | | | |
|---|---|---|---|---|---|
| Cancer cervix is a curse (very bad something) | ☐ | ☐ | ☐ | ☐ | ☐ |
| Screening causes harm to the client | ☐ | ☐ | ☐ | ☐ | ☐ |
| screening for cervical cancer is expensive | ☐ | ☐ | ☐ | ☐ | ☐ |
| **Item related to pap smear** | **Yes** | | **No** | | |
| **What is the proper age at which Pap smear test should be started?** | | | | | |
| From puberty | ☐ | | ☐ | | |
| From 20 years | ☐ | | ☐ | | |
| From 30 years | ☐ | | ☐ | | |
| After menopause | ☐ | | ☐ | | |
| I don't know | ☐ | | ☐ | | |
| **Best time for doing Pap smear test:** | | | | | |
| During menstrual flow | ☐ | | ☐ | | |
| A week after period | ☐ | | ☐ | | |
| Not sure | ☐ | | ☐ | | |
| **Pap smear test should be done by:** | | | | | |
| Any physician | ☐ | | ☐ | | |
| Gynecologist | ☐ | | ☐ | | |
| The 1st two | ☐ | | ☐ | | |
| Trained nurse | ☐ | | ☐ | | |
| Not sure | ☐ | | ☐ | | |
| **What is the proper interval for doing Pap smear test?** | | | | | |
| Monthly | ☐ | | ☐ | | |
| Yearly | ☐ | | ☐ | | |
| After menopause | ☐ | | ☐ | | |
| Not sure | ☐ | | ☐ | | |
| **If there is abnormality in Pap smear test results, what should be done?** | | | | | |
| Leave it to God and pray | ☐ | | ☐ | | |
| Do confirmatory lab tests | ☐ | | ☐ | | |
| Not sure | ☐ | | ☐ | | |
| **What are the benefits of Pap smear test?** | | | | | |
| Detection of any early abnormal changes in the cervix | ☐ | | ☐ | | |
| Early detection of cervical cancer | ☐ | | ☐ | | |
| The 1st two items | ☐ | | ☐ | | |
| Not sure | ☐ | | ☐ | | |
| **Pap smear test is done using:** | | | | | |
| Transvaginal ultrasound | ☐ | | ☐ | | |
| Vaginal brushing | ☐ | | ☐ | | |
| Not sure | ☐ | | ☐ | | |
| Others | ☐ | | ☐ | | |

**Section IV: Preventive practices and potential barriers against cervical dysplasia,** *Kindly tick the appropriate response according to your knowledge for the following items;*

**Have you ever vaccinated against human papillomavirus?** ☐No ☐Yes

**Have you undergone pap smear test?** ☐No ☐Yes

**If yes; what were the test results?** □normal findings □not done □ susceptible or positive

**If yes; when was the last scan time?**

□One year ago. □≥ two years ago □Not done

**What are the reasons for doing the tests?**

□General health checks up □Doctor recommendation

□Complain from itching, bleeding, discharge, etc □Not done

| **Barriers to CC screening** | **Yes** | **No** | **I don't know** |
|---|---|---|---|
| ***Psychosocial barriers*** | | | |
| Does your culture forbid cervical screening? | □ | □ | |
| No need so far there is no complaint | □ | □ | |
| Is CC screening embarrassing? | □ | □ | |
| Is cervical cancer screening painful? | □ | □ | |
| Screening is not helpful to prevent God willing | □ | □ | |
| Fear of positive finding | □ | □ | |
| ***Socioeconomic barriers*** | | | |
| Screening test is expensive | □ | □ | □ |
| I don't have enough time for screening | □ | □ | □ |
| Screening test isn't available in my workplace | □ | □ | □ |
| My husband might not agree to do screening | □ | □ | □ |
| ***Healthcare system barriers*** | | | |
| There is long waiting time at health facility to do screening | □ | □ | □ |
| I don't know which health facility offering cancer cervix screening | □ | □ | □ |
| I don't know which health facility offering cancer cervix screening | □ | □ | □ |
| I do not know whom to consult for undergoing this test | □ | □ | □ |

***End of the questionnaire, kindly submit your response.***

***Thank you for your contribution in the study .***
